# PPI Long Term Use: Risk of Neurological Adverse Events?

**DOI:** 10.3389/fneur.2018.01142

**Published:** 2019-01-08

**Authors:** Michal Novotny, Blanka Klimova, Martin Valis

**Affiliations:** ^1^Biomedical Research Centrum, University Hospital Hradec Králové, Hradec Králové, Czechia; ^2^Department of Neurology, University Hospital Hradec Kralove, Hradec Králové, Czechia

**Keywords:** dementia, cognitive disorders, affective disorders, depression, proton pump inhibitors, omeprazole, acidity, reflux disease

## Abstract

The purpose of this review study is to reveal a potential threat of one type of such widely used and freely distributed drugs, which are proton pump inhibitors that might be the cause of the onset of both dementia and depression. The authors performed a literature review of available studies on the research topic describing the adverse effect of proton pum inhibitors (PPIs) (omeprazole, esomeprazole, lansoprazole, dexlansoprazole, rabeprazole, pantoprazole, dexrabeprazole, ilaprazole). For a long time, PPIs were considered to be completely safe drug substances for both short and long-term use. In recent years, there have been a few contradictory studis of absolute safety, especially in patients, who have long been using PPIs. At this time when depression and dementia are rising in the population, this is a very worrying fact that needs to be highlighted, and which needs to be carefully studied and evaluated, ideally trying to prevent it. The findings of most research studies described in this review indicate that there is a direct association between the onset of dementia and depression on one side and the long-term use of PPIs on the other.

## Introduction

As a result of the rise in the number of aging population groups worldwide, there is an increase in the number of aging diseases. Particularly, mental and neurological disorders among older adults form 6.6% of the total disability, out of which dementia and depression, are the most common and devastating. They affect approximately between 5 and 7% of the world's older population (1). This raises other issues such as an economic and social burden (2). As Langa (3) reports, the economic effect of dementia is much bigger than with chronic diseases (i.e., cancer or heart attack). It reaches about 600 billion US dollars worldwide. Similarly, depression is considered to be a major public health problem. According to WHO (4), depression is the fourth common cause of morbidity. In fact, by 2020 it is supposed to reach the first place. It affects around 22% of males and 28% of females at the age of 65 years and over. In addition, it is estimated that 85% of older people with depression receive no help at all from the National Health Service (5). It is estimated that the global cost of mental health problems forms 2.5 trillion US dollars, which is again greater than with cardiovascular diseases, chronic respiratory diseases, cancer and diabetes (6).

Therefore, there is effort to prevent these mental and neurological disorders in order to reduce economic burden and maintain the quality of life of these elderly people. Currently, there is no reliable cure for dementia (7) and depression (8). Nevertheless, there are several strategies such as psychosocial interventions and drug administration which can help in the treatment of their symptoms. In comparison with psychosocial interventions, there are many different widely used drugs, for example, antidepressants which can have many side effects such as nauzea, increased appetite and weight gain, loss of sexual desire and other sexual problems such as erectile dysfunction and decreased orgasm, fatigue and drowsiness, or insomnia (9). The same is true for dementia. At present, there have been only four clinically approved drugs (i.e., donepezil, galantamine, memantine, and rivastigmine) which can help patients to delay the process of the disease for some time, but they are quite expensive, may have negative side effects and they do not quite help people to improve their physical or social conditions (10).

The purpose of this review study is to reveal a potential threat of one type of such widely used and freely distributed drugs, which are proton pump inhibitors that might be the cause of the onset of both dementia and depression.

## Methods

The authors performed a literature review of available studies on the research topic describing the adverse effect of proton pum inhibitors (PPIs) (omeprazole, esomeprazole, lansoprazole, dexlansoprazole, rabeprazole, pantoprazole, dexrabeprazole, ilaprazole). This review focuses on the studies, whose neurological side effects (affective disorders and cognitive disorders) were detected after the administration of PPI. The research studies were selected on the basis of research topics. The key search collocations/words were *neurological adverse effect, dementia, depression, proton pump inhibitors (PPI)*, and *treatment* found in the world's acknowledged databases Web of Science, PubMed, Springer and Scopus. The search was not limited by any period. These research studies were classified according to their relevancy. Eventually, 54 studies, including the web pages, were involved in final analysis. The information found in the selected studies on dementia, depression, neurological adverse effect in connection with PPI use was carefully evaluated and it is described and discussed in the following sections.

### Proton Pump Inhibitors

Proton pump inhibitors are among one of the top groups of active substances (11, 12). They are available both on prescription and over-the-counter. Expenditure on these prescription drugs was $13 billion worldwide in 2009. This amount did not include PPI sold freely in the pharmacy (OTC) (13). Moreover, with the rising consumption of drugs, it is expected that the amount will be now even higher.

The name of this group is derived from their mechanism of action. The highly specialized transport system—the proton pump—is responsible for stomach acid production. By replacing the potassium ions (K^+^), it releases hydrogen protons (H^+^) and results in secretion of hydrochloric acid. A PPI substance covalently attaches to this pump, irreversibly inhibits it, and secretion of HCl ceases. The secretion activity can be restored only by *de novo* proton pump synthesis, but not earlier than within 24 h. The advantage of PPI is that it suppresses gastric acid secretion independently of the origin of the stimulus and is thus universally usable (14, 15).

The first approved molecule for use in clinical practice was omeprazole in 1989 (USA, GB). Then other molecules (Table 1) followed (in different countries of the world independently of each other) such as esomeprazole, lansoprazole, dexlansoprazole, rabeprazole, pantoprazole, dexrabeprazole, ilaprazole (15).

**Table 1:** Overview of all the world's traded PPIs (in the period of 2016–2018) and their evipotent doses (mg) for oral administration.

**Molecule**	**WHO (16)**^**a**^	**WGO global guidelines (17)**^**b**^	**NICE guidance (18)**^**c**^
Pantoprazole	40	40	40/40
Omeprazole	20	20	20/40
Esomeprazole	30	40	20/40
Rabeprazole	20	20	20/20
Lansoprazole	30	30	30/30
Dexlansoprazole	30	60	N/A
Ilaprazole^*^	This molecule is currently only traded in China, India, and Korea. The recommended daily dose for adult therapy in the indication of GERD, dyspepsia and peptic ulcers ranges from 5 to 20 mg.		
Dexrabeprazole^**^	This molecule is currently only traded in Turkey and India. The recommended daily dose for the treatment of adults with gastro-esophageal reflux disease, gastric and duodenal ulcers ranges from 5 to10 mg.		
S-pantoprazole^***^	This molecule is currently only traded in India, Korea, Colombia and the Dominican Republic. The recommended daily dose could not be traced		

*Not all PPIs may be available in all countries (the standard PPIs may differ in some countries)*.

a *The defined daily doses (DDD) are based on the treatment of gastro-oesophageal reflux disease*.

b *Recommended daily dosage for healing in erosive esophagitis*.

c *Recommended daily dosing is given in the recommendations in the form of: Gastro-oesophageal reflux disease and dyspepsia in adults / doses for severe oesophagitis*.

* *WHO/WGO/NICE dosing was not established. The data were taken from the Ilacare® product summary*.

** *WHO/WGO/NICE dosing was not established. Data were derived from the Dirab® product summary*.

*** *WHO/WGO/NICE dosing was not established. Valid information on indications and dosage has not been found for Panpure® and Leptopra®*.

The use of PPI within approved indications is quite broad: treatment of gastric and duodenal ulcers and esophageal reflux disease. PPIs are also part of eradication combinations; they serve to prevent upper gastrointestinal bleeding when administered with a non-steroidal anti-inflammatory drug (NSAID). Barrett's esophagus and hypersecretion syndromes (e.g., Zollinger-Ellison syndrome) are less common indications (15, 19). Despite this range of indications, according to available data, for example, from Italy, 40–60% of PPIs are prescribed inadequately and outside the designated indications (12, 20). The same trend can be expected worldwide, i.e., PPIs are overused in particular in unapproved indications (e.g., dyspepsia, vague digestive disorders, or food-related difficulties). In addition, because some of these products are OTC, there is no medical check about the amount of free PPI preparations.

For a long time, PPIs were considered to be completely safe drug substances for both short and long-term use. In recent years, there have been contradictory studies of absolute safety, especially in patients who have long been using PPI (15). In addition to the undesirable effects that are easily solved, references to the long-term relationship of PPIs and neurological problems have emerged (12, 19). At this time when depression and dementia are rising in the population, this is a very worrying fact that needs to be highlighted, and which needs to be carefully studied and evaluated, ideally trying to prevent it.

### Proton Pump Inhibitors—Side Effects

PPIs have a very good safety profile for short-term administration (Table 2). However, this can no longer be assured with increasing frequency of long-term administration (Table 2), which is becoming commonplace for different types of indicated patients (24). This section provides a brief overview of current evidence and views on this drug group, particularly in terms of potential side effects, which stimulated professional discussion on vitamin B_12_ deficiency, iron deficiency, hypomagnesaemia, bone fractures, intestinal infections and adverse effects on affectivity, and cognition (24, 25). The last two named units (i.e., depression and dementia) are described in more detail in this article.

**Table 2:** Adverse events (AE) reported in patients treated with PPI (21, 22).

**The commonest AEs (short-term use)**	**AEs unrelated to acid inhibition (long-term use)**	**AEs related to acid inhibition** **(long-term use)**
Headache Nausea Diarrhoea Abdominal pain Constipation Dizziness Fatigue Rash Pruritus	Allergic reaction to drug chemicals Collagenous colitis Acute interstitial nephritis Chronic kidney disease Drug interaction Dementia Cerebral ischemic diseases Ischemic cardiac diseases	Pneumonia Gastrointestinal infection Gastric carcinoid tumor Gastric fundic mucosal hypertrophy Changes in gut microbiome Small intestinal bacterial overgrowth Iron deficiency Bone fracture Vitamin B12 deficiency Hypomagnesemia Gastric fundic gland polyps Gastric cancer Colon cancer Spontaneous bacterial peritonitis Hepatic encephalopathy Drug interaction

*The basic analysis of reported adverse drug, sorted according to a total number of reports, age group, sex, geographic region from EUDRAVIGILANCE (European database of suspected adverse drug reaction reports) can be found in Table 3*.

**Table 3:** Basic analysis of adverse drug (sorted according to a total number of reports, age group, sex, geographic region).

	**PAN**	**OME**	**ESO**	**RAB**	**LAN**	**DEXLAN**
Total Number of Cases	20.287	30.647	28.469	5.620	19.296	6.101
**AGE GROUP**						
Not specified	8.202	9.828	9.538	2.503	9.468	5.425
0–1 months	33	66	38	5	14	0
2 months−2 years	38	190	116	7	98	0
3–11 years	49	187	92	3	75	1
12–17 years	83	273	113	15	86	4
18–64 years	6.326	10.662	10.294	I.51	4.584	428
65–85 years	4.651	7.828	6.796	1.368	4.138	219
More than 85 years	905	1.613	1.482	209	833	24
**SEX**						
Female	9.023	14.272	13.875	2.071	7.286	1.292
Male	6.405	10.456	8.663	1.578	5.287	803
Not Specified	4.859	5.919	5.931	1.971	6.723	4.006
**GEOGRAPHIC ORIGIN**						
EU economic area	8.151	9.673	7.111	1.279	3.651	22
Non-EU economic area	12.133	20.971	21.357	4.341	15.644	6.079
Not specified	3	3	1	0	1	0

*Source: EUDRAVIGILANCE, European database of suspected adverse drug reaction reports (23); PAN, Pantoprazole; OME, Omeprazole; ESO, Esomeprazole; RAB, Rabeprazole; LAN, Lansoprazole; DEXLAN, Dexlansoprazole; Ilaprazole, dexrabepratole, and S-pantoprazole are not in the EU database*.

The presence of HCI in the stomach is essential for the **absorption of vitamin B**_**12**_ and therefore a number of studies have been conducted to demonstrate or counteract the negative effect of PPI on blood levels of vitamin B_12_. It has been found that a healthy eating population does not occur with commonly used PPI doses to B_12_ deficiency. B_12_ deficiency is at risk for seniors and long-term patients with nutrition disorders that have a 2 to 4-fold increased risk of vitamin B_12_ deficiency (26). For completeness, it should be added that there is an older study not demonstrating this risk (27).

Stomach HCl also facilitates the absorption of iron salts contained in food or in conventional iron-containing medicines. The results of two studies by Sarzynski et al. (28) and Ajmera et al. (29) show that there is a need to think about the possibility of reducing iron absorption and that this risk group is represented by seniors. In the first study, the mean age of patients was 56 years, and it was found that even after one year of PPI use, there was a significant decrease in blood hemoglobin and hematocrit. In the second study, patients with hypochromic anemia with an average age of 50 years received omeprazole over the long term. The results showed that there was an inadequate response to oral anemia treatment with iron observed in 84% cases. In this case, it should be noted that there is a study by Stewart et al. (30), in which there was no evidence of iron deficiency or even a decrease in total iron stores in patients with Zollinger-Elison syndrome using 6 years of PPI.

Rare, but potentially very dangerous, hypomagnesaemia—a **magnesium deficiency**, has been described as an undesirable effect of PPI treatment in several dozen case reports since 2006 (31). Patients have experienced a deficiency in the continuous, at least one–year use of PPI. It is not yet known why this occurs in some patients. It is known that the decline occurs gradually after the discontinuation of PPI reversible (normalization for 1–2 weeks), but with the re-deployment of PPI, the deficit is again manifested (roughly in days) (32).

Evidence of PPIs for **bone mineralization and bone fractures** is not yet consistent, however, positive associations predominating for the PPI association and the risk of fractures predominate. Yang et al. (33) showed an increased risk only in patients over 65 years of age. Vestergaard et al. (34) confirmed these results. Targownik et al. (35) found only the risk of more than 6 years of PPI administration. On the contrary, Corley et al. (36) did not find any statistically significant risk in the general population. In spite of obvious contradictions in medical literature, the US State Authority FDA responded in 2010 and ordered updating information on PPI-containing medicines. Medicines should therefore include a warning that the risk of fractures of the proximal end of the femur, distal end of the forearm and vertebrae may be increased by PPI.

Another reported side effect associated with long-term PPI treatment is an **increased risk of infections**. Infections of both small intestinal bacterial overgowth (SIBO), non-typhoidal salmonella and campylobacter infections, clostridium difficile infections (CDI) and total (pneumonia). The relationship between the use of PPIs and the development of SIBO does not seem to doubt the experts. The only difference in the study is the mismatch between the rate of increased reactive risk. If researchers use the aspirin analysis to identify SIB, the risk of SIBO is 6- to 20-fold (37, 38), whereas when a breath test is used, the risk rate is only 2 times bigger (39). In the context of the development of salmonella or campylobacter infection, the study by Bavishi and Dupont (40) indicates a 3-fold increased risk of intestinal infection in PPI therapy. Patients using PPI have been shown to have a higher risk of diarrhea caused by clostridium difficile by 74–136%. A higher risk has been demonstrated in the elderly, in patients treated with ATB and hygiene neglect (41).

### Proton Pump Inhibitors—A Risk of Neurological Adverse Events?

For the first time in 1997, Fireman et al. (42) reported three cases of severe central nervous system disorder during the treatment with omeprazole, but which had disappeared after discontinuation. Since then, PPI has been monitored for potential neurological side effects.

#### Dementia

Since Fireman et al. (42) published their report, more than 20 years have passed and several original publications on cognitive impairment and PPI use have been written (19, 43–45). Most recently, a summary review (46) has been published. This topic still remains full of controversy (47, 48). The overview and specifications of individual clinical trials evaluating the effect of PPI on the potential development of neurological adverse events are provided in Table 4 below.

**Table 4:** Overview and specifications of individual clinical trials evaluating the effect of PPI on the potential development of neurological adverse events.

**References**	**Type of study**	**Number of patients**	**Type of PPI/dosage if is available (mg/day)**	**Duration of treatment**	**Observed neurological AE**
Haenisch et al. (44)	Multicenter cohort	3.327	Omeprazole, esomeprazole, lansoprazole, pantoprazole, rabeprazole, dexlansoprazole	18 months	Significantly increased risk of any dementia
Akter et al. (43)	Randomizovaná, placebem kontrolovaná	60	Omeprazole (42), lansoprazole (49), rabeprazole (39), pantoprazole (42), esomeprazole (42)	1 week	Impairment in visual memory, attention, executive function, and working and planning function
Gomm et al. (19)	Prospective cohort	73.679	Omeprazole, pantoprazole, lansoprazole, esomeprazole, rabeprazole	7 years	Significantly increased risk of incident dementia
Tai et al. (45)	Multicenter cohort	15.726	N/A (subgroup analysis: omeprazole, pantoprazole, and lansoprazole)	2 years	Increased risk for dementia
Booker et al. (47)	Case-control	23.912	N/A	4 years	Decreased risk of developing dementia
Golstein et al. (48)	Observational, longitudinal	10.486	Omeprazole, omeprazole–sodium bicarbonate, esomeprazole, lansoprazole, pantoprazole, rabeprazole, dexlansoprazole	10 years	No association with greater risk of dementia or of ad
Galati et al. (50)	Multicenter cohort	179.786	Omeprazole, esomeprazole, rabeprazole, lansoprazole	2 years	High prevalence of neuropsychiatric reactions related to proton pump inhibitors
Polimeni et al. (51)	Case report	1	Rabeprazole	10 days	Anxiety, panic attacks, pavor nocturnus, episodic mental confusion, attention deficit
Laudisio et al. (12)	Population-based study	344	Omeprazole (39), esomeprazole (42), lansoprazole (49), rabeprazole (39), pantoprazole (39)	N/A	Increased probability of depression
Abela et al. (52)	Case report	1	Omeprazole (80 i.v.)	12 hours	Delirium
Heckmann et al. (53)	Case study	1	Omeprazole (80 i.v.)	5 days	Psychotic symptoms
Otremba et al. (49)	Prospective cross-sectional	675	N/A	N/A	Delirium

Badiola and his team were the first to publish in 2013 a study on a possible association of lansoprazole (transferred to other PPIs) about an increased production of amyloid beta (Aβ), not only in cell cultures but also in animal models Badiola et al. (54). Although the exact mechanism remains unknown, the authors suggest that lansoprazole increased levels of Aβ37, Aβ40, Aβ42, and also lowered the level of Aβ38 in AD models. In addition, lansoprazole appears to have also influenced the enzyme activity of β-,γ-secretases, the substances necessary to cleave the amyloid precursor protein from which toxic Aβ is formed. In animal models, lansoprazole promoted an increase in Aβ40 levels, which could indicate that lansoprazole may also *in vivo* impair the production of Aβ.

A study of another type, but also pointing to a possible link with dementia, was published by Haenisch et al. (44). This is an epidemiological study of data from a longitudinal, multicenter, cohort study on elderly patients in primary care in Germany. The aim was to evaluate a possible association between the use of PPI and the risk of dementia in the elderly. Overall, 3,327 people aged 75 years and over were included in the survey. During the 18-month period, the authors found a total of 431 patients with dementia (without species determination), including 260 Alzheimer's disease patients. Using advanced statistical methods, patients with PPI had a significantly increased risk of dementia (HR = 1.38, 95% CI 1.04–1.83) and Alzheimer's disease (HR = 1.44, 95% CI 1.01–2.06) with those who did not use PPIs. According to the authors, avoiding the use of PPIs in the elderly individuals could be an essential element of preventing dementia.

In light of previous publications, Akter et al. (43) decided to place a placebo-controlled experiment with 60 healthy young volunteers (aged 20–26 years, both sexes equally) to assess the impact of 5 specific PPIs (omeprazole, lansoprazole, rabeprazole, pantoprazole, esomeprazole) on various cognitive functions. Testing was carried out using the Cambridge Neuropsychological Test Automated Battery (CANTAB) software, in which 5 parts (Motor Screening Test, Paired Associates Learning, rapid visual information processing and spatial working memory) were selected for this purpose. Because some sections had more subparts, the total number of tests was 9. Volunteers completed this test before PPI administration and after 7 days of PPI use. Surprisingly, it was found out that all PPIs showed a similar negative effect on cognitive function. A significant decrease (*p* < 0.05) was observed for omeprazole in 7 tests for lansoprazole and pantoprazole at 5, for rabeprazole at 4. Esomeprazole appeared in group 5 PPI best and significantly influenced cognition in only 3 tests. Even though this pilot study with PPI has its shortcomings (e.g., a small study sample), it provides a warning signal to doctors and pharmacists who should consider the possible risks and benefits of prescribing PPI.

The second in the sequence, who sought to find the association between the PPI administration and the increased risk of cognitive defect in old age, and in a larger number of patients, was Gomm et al. (19). In 2016, they published a prospective cohort study based on patient data from the largest German Health Insurance Company (AOK). For the purpose of the investigations, the period between 2004 and 2011 was used. For the purposes of diagnosis, the codification was used by the German Statistical Classification of Diseases and Related Health Problems (Tenth Revision) and as pharmaceuticals of interest, the following were chosen: omeprazole, pantoprazole, lansoprazole, esomeprazole, or rabeprazole. By using time-dependent Cox regression, the authors tried to eliminate possible other causes of dementia such as age, gender, comorbidity, or polypragmia. Altogether, 73,679 patients over 75 years of age, who had no dementia at any given start, i.e., in 2004, were examined. As a result, a group of patients who had long-term PPI (*n* = 2,950; mean [SD] age, 83.8 [5.4] years, 77.9% female) compared to patients who did not use PPI (*n* = 70 729; mean [SD] age, 83.0 [5.6] years; 73.6%female) had a significantly increased risk of dementia (HR = 1.44 [95% CI, 1.36–1.52]; *P* < 0.001). The authors conclude that the results are in line with previous published study and that avoiding the use of PPIs at risk patients could prevent them from developing dementia.

Based on published concerns about the possible association between the PPI use and dementia development, a team of Taiwanese scientists (45) decided to carry out an epidemiological assessment of the Asian population. The data was obtained from Taiwan's National Health Insurance Research Database between 2000 and 2003. Overall, 15,726 patients over 40 years of age were diagnosed without dementia at baseline. By advanced statistical method (Cox regression), it was discovered that patients using PPI (*n* = 7,863; mean follow-up 8.44 years) were significantly more at risk of developing dementia than those without PPI medication (*n* = 7,863; mean follow-up 8.44 years). Results were published in adjusted HR = 1.22; 95% CI: 1.05–1.42. By analyzing subgroups that were not published by anyone, the authors concluded that the risk of dementia in PPI patients was increased by co-morbidities such as depression (adjusted HR = 2.73, 95% CI: 1.91–3.89), hyperlipidemia (adjusted HR = 1.81; 95% CI: 1.38–2.38), ischemic heart disease (adjusted HR = 1.55; 95% CI: 1.12-2.14) and hypertension (adjusted HR = 1.54: 95% CI: 1.21–1.95). The authors concluded that the possible association between the use of PPI and dementia had been identified, and the mechanism of how this could be done remains unclear.

The opposite view on this issue was provided by the study of Booker et al. (47). The aim of their study was to identify the risk factors for dementia in German primary care patients. Epidemiological follow-up included patients aged 70–90 years with a first diagnosis of dementia for any cause (*n* = 11,956) between 2010 and 2014. The study included a control group without dementia (*n* = 11,956) in a ratio of about 1:1 in terms of age, gender, type of insurance and doctor. Patient records from up to 10 years prior to diagnosis of dementia were monitored. Statistical methods found that factors that significantly increased the risk of dementia included diabetes, lipid metabolism disorders, stroke, Parkinson's disease, intracranial damage, coronary artery disease, mild cognitive deficits, and mental or behavioral disorders caused by alcohol. Quite differently from previously published results, the authors did not find any association between the PPI use and dementia (OR = 0.93; 95% CI: 0.90–0.97). Values rather indicate a slight decrease in the risk of dementia. The authors are aware of the differences in their results and therefore propose to conduct further studies while highlighting some of the shortcomings that their study has.

Furthermore, Goldstein et al. (48) evaluated the effect of PPI on the development of mild cognitive deficits, dementia, and especially Alzheimer's disease. This was a longitudinal observational study conducted at Alzheimer's disease therapy centers (Tertiary Specialized Centers). Their study included volunteers aged 50 years or older who did not have a cognitive impairment at baseline or had a mild cognitive impairment. 884 patients reported a regular long-term use of PPI, 1,925 intermittent PPI use and 7,677 reported that PPI had never been used. With the continued use of PPI (compared with the use of type *never*), the risk of dementia (HR = 0.78, 95% CI 0.66–0.93), or the risk of moderate cognitive impairment in Alzheimer's dementia (HR = 0.82, 95% CI = 0.69–0.98) were not found. The same negative results were obtained by the authors in the evaluation of patients with intermittent administration of PPI. The authors conclude that PPIs should not be associated with an increased risk of dementia or Alzheimer's disease, but the authors point out that the greatest weakness may be simple reporting by patients themselves and no drug delivery control.

The situation with regard to the inexperience in terms of PPI use and the possible risk of developing dementia was summarized in a systematic review by Batchelor et al. (46). They evaluated all the studies published in the following databases: MEDLINE, EMBASE, Cochrane Central Register of Controlled Trials, PSYCinfo, Scopus, Web of Science, and ClinicalTrials.gov until June 2016. The review was conducted in a standardized manner, including all types of studies. Patients under the age of 18 years were excluded. Two independent reviewers evaluated the quality and data of entry studies. Finally, 11 articles were included in the study, of which the effect of PPI and dementia was studied in four studies. The relationship between PPI and immediate cognitive impairment was addressed in seven studies. Three of the four papers showed a positive correlation between PPI and dementia. In other studies, the relationship between PPI and the immediate reduction of cognitive function was observed. The authors concluded that each published study had some level of uncertainty and risk of bias. In addition to a poorly selected design, a small sample of subjects, an unclear dementia definition/classification, studies may also fail to take into account other important factors that affect dementia development.

#### Affectivity Disorders

Proton pump inhibitors also do not avoid reports of possible neuropsychiatric influences. Galati et al. (50) published a study that attempted to map out the occurrence of drug-induced non-psychiatric illnesses or undesirable effects in general practitioners' surgeries. Data from the study was taken from an Italian database recording a spontaneous reporting of adverse drug reactions by GPs. Between 2002 and 2003, 171 doctors reported 1,131 side effects, of which 440 were CNS (241 neurological and 199 psychiatric). For the purpose of the study, 40 reports were excluded for their incompleteness. In the study, there is evidence that 10 reports were received for esomeprazole and three reports for rabeprazole. Disorders reported include abnormal thinking, hallucinations, and hyperkinesia.

In 2007, Polimeni et al. (51) published a case report of 55-year-old patient who was prescribed rabeprazole (20 mg daily in the morning) for dyspeptic complaints. Ten days later, the patient described fear, panic attacks, night terrors, episodic confusion, and disorder. After the discontinuation of PPI, the patient's status within 2 days was normalized. Subsequently, the patient was prescribed esomeprazole and the neuropsychiatric problems did not appear anymore. The authors considered the association of PPI induced hypersecretion of gastrin, of which rabeprazole had the strongest of all PPI expressed and psychiatric problems.

The situation in the field of neuropsychiatric undesirable effects (specifically depression) after the use of PPI in the elderly population was described by Laudisio et al. (12). With 304 Italian seniors over the age of 75 years, the 30-item Geriatric Depression Scale (GDS) was filled in, with depression being defined at 11 or more. As a result, the finding was that the PPI use was associated with a significantly higher probability of depression (OR = 2.38; 95% CI = 1.02–5.58). In addition, higher doses of PPI were found to show a higher probability of depression. Conversely, no association was detected with antacids or H_2_ blockers. The authors found out that 14% of the depression could be avoided by excluding PPI. The authors concluded that the use of PPI could become episodic disorders in elderly people and therefore suggested that mood should be routinely evaluated in elderly people using PPI.

For the sake of completeness, it should be noted that the published literature also contains references to the occurrence of delirium in PPI administration, but it is more about case reports rather than studies on a larger number of patients. However, the data differs even in this respect. Some articles testify the possibility of delirium after PPI (49, 52, 53) and some deny it (55).

## Discussion and Conclusion

The findings of most research studies described above indicate that there is a direct association between the onset of dementia and depression on one side and the long-term use of PPIs on the other.

Although the mechanism of action of the PPI use in dementia is not that clear, one of the factors can be deficiency of vitamin B_12_. Lam et al. (26) argue that the long-term use of PPI suppresses the production of gastric acid and thus contributes to malabsorption of vitamin B_12_. In their study with large sample sizes, they showed that patients who were administered PPIs for two or more years (OR, 1.65 [95% CI, 1.58–1.73]) had been associated with an increased risk for vitamin B_12_ deficiency. In addition, Fallahzadeh et al. (56) indicate that chronic consumption of PPIs may act as a risk factor for the onset of Alzheimer's disease since PPIs may basify lysosmes and hamper degration of fA beta, which is one of the major events in the pathogenesis of Alzheimer's disease.

The findings about the association of the PPI use and the onset of dementia were also confirmed in a systematic review by Batchelor et al. (46). However, due to the heterogeneity of the obtained data and different methodologies, their meta-analysis could not be conducted. Therefore, their results might lead to the overestimated effects. In conclusion, they suggest performing risk-benefit analysis when prescribing PPI, especially for older people.

In conclusion, it should be added that the PPI therapy is not the only existing solution. If a physician or pharmacist considers that the PPI use is inappropriate for a particular patient or needs to be stopped (for adverse reactions), s/he may choose from a variety of other gastroenterological products with minimal risk to the central nervous system. S/he must be always guided by the severity and nature of the reported symptoms. At the same time, with alternative prescriptions, the patient should be advised about a change in lifestyle (e.g., limit smoking or stop it, weight reduction, regular exercise, wear loose clothing, eat 5 to 6 small portions food, or sleep on the left side). Antacids are OTC substances that alleviate gastric acid overdose, but the duration of their action is very limited. Another alternative could be H2 blockers (ranitidine, famotidine), where more frequent dosing (up to 3 times a day) is needed. Another way of treatment could be to strengthen the protective mucosal layer by the so-called mucoprotective agents (sucralfate, misoprostol, bismuth-subsalicylate). A brand new generation of substances is Japan's approved prazans (vonoprazan). The latest possible alternative to PPI may be surgical intervention, e.g., various types of fundoplications.

## Author Contributions

MN, BK, and MV equally contributed to the drafting, analyses and final version of the whole manuscript. All authors read and approved the final manuscript.

### Conflict of Interest Statement

The authors declare that the research was conducted in the absence of any commercial or financial relationships that could be construed as a potential conflict of interest.
